# Determination of Chlortetracycline Residues, Antimicrobial Activity and Presence of Resistance Genes in Droppings of Experimentally Treated Broiler Chickens

**DOI:** 10.3390/molecules23061264

**Published:** 2018-05-25

**Authors:** Javiera Cornejo, Karina Yevenes, Constanza Avello, Ekaterina Pokrant, Aldo Maddaleno, Betty San Martin, Lisette Lapierre

**Affiliations:** 1 Department of Preventive Medicine, Faculty of Veterinary and Animal Sciences, University of Chile, Av. Santa Rosa, La Pintana, Santiago 11735, Chile; kariyevenescoa@gmail.com (K.Y.); coniavello@gmail.com (C.A.); katiavalerievna@ug.uchile.cl (E.P.); amaddaleno@veterinaria.uchile.cl (A.M.); 2 Laboratory of Veterinary Pharmacology, Faculty of Veterinary and Animal Sciences, University of Chile, Av. Santa Rosa, La Pintana, Santiago 11735, Chile; bsmartin@uchile.cl

**Keywords:** tetracycline residues, depletion, poultry droppings, antimicrobial activity, antimicrobial resistance genes

## Abstract

Tetracyclines are important antimicrobial drugs for poultry farming that are actively excreted via feces and urine. Droppings are one of the main components in broiler bedding, which is commonly used as an organic fertilizer. Therefore, bedding becomes an unintended carrier of antimicrobial residues into the environment and may pose a highly significant threat to public health. For this depletion study, 60 broiler chickens were treated with 20% chlortetracycline (CTC) under therapeutic conditions. Concentrations of CTC and 4-epi-CTC were then determined in their droppings. Additionally, this work also aimed to detect the antimicrobial activity of these droppings and the phenotypic susceptibility to tetracycline in *E. coli* isolates, as well as the presence of *tet(A)*, *tet(B)*, and *tet(G)* resistance genes. CTC and 4-epi-CTC concentrations that were found ranged from 179.5 to 665.8 µg/kg. Based on these data, the depletion time for chicken droppings was calculated and set at 69 days. All samples presented antimicrobial activity, and a resistance to tetracyclines was found in bacterial strains that were isolated from these samples. Resistance genes *tet(A)* and *tet(B)* were also found in these samples.

## 1. Introduction

Antimicrobial drugs have a prominent role within the veterinary and animal sciences as they have been used not only for therapeutic and preventive purposes, but also as growth promoters [1]. Although the European Union has banned the latter practice, it is unfortunately still common in other countries [2,3].

Tetracyclines are the main antimicrobial drugs currently in use for veterinary purposes. Sulfonamides and macrolides are the next most common drugs. Altogether, these classes comprise approximately 90% of all antimicrobials used in the United Kingdom and close to 50% in South Korea [4].

This class of antimicrobials shows bacteriostatic activity against a wide array of Gram-positive and Gram-negative bacteria, mycoplasmas, some mycobacteria, as well as several protozoa and filariae too [5]. In particular, chlortetracycline (CTC), tetracycline (TC), oxytetracycline (OTC), and doxycycline (DXC) are the most commonly used drugs in poultry farming operations [6,7]. Furthermore, the extent of their use within the poultry industry makes it unlikely to imagine its sustainability in the absence of these antimicrobials, despite the debates and controversies regarding the possible consequences to public health that might derive from the use of antimicrobials in animal farms [8].

According to Massé et al. [9], animals excrete between 17% and 90% of a single dose of antimicrobials following its administration. Antimicrobial drugs are eliminated either as active metabolites (epimers or isomers) or in their original form. As for tetracyclines, more than 70% are excreted and released into the environment in their active form via urine and feces in both humans and animals [10]. Therefore, high concentrations of tetracyclines are likely present in feces. In this regard, several researchers have found that this exact scenario also occurs in bird droppings [11,12,13,14,15,16]. These studies also found that OTC, CTC, and DXC were the most prominently represented drugs in this matrix, and their concentrations averaged from 0.01–186 mg/kg [15].

Similarly to other tetracyclines, CTC is not metabolized [17]. Under fairly acidic conditions, this drug can reversibly form the 4-epi-chlortetracycline epimer, which is also a degradation product of CTC. This epimer shows less antimicrobial activity but closely resembles the behavior of CTC regarding how it creates complexes with several components of manure, such as metal ions, humic acids, proteins, and organic matter. Such complexes strongly bind this epimer to this matrix [9,18].

Broiler beds are a byproduct from the poultry industry that comprises not only bedding materials, but also bird droppings that are spilled over food [19]. Worryingly, some antimicrobials have been reported in this material [20,21]. According to some estimations, the poultry industry in Chile produces up to 200,612 metric tons of these beds every year. Therefore, the presence of antimicrobial drugs in them becomes a significant concern [22].

As broiler beds have become organic fertilizers that are used by farmers throughout the world [1,19,23,24,25], they pose a serious risk to public health because these fertilizers are one of the main routes that allow for the spillover of antimicrobials in the environment [26]. Furthermore, this practice also spreads bacteria that are already resistant to antimicrobials, as well as antimicrobial resistance genes (ARGs) and mobile genetic elements that may carry resistance genes over, such as plasmids and integrons [24,27]. All of these elements are closely linked to broiler chicken bedding. In fact, the evidence shows that the prevalence of antimicrobial resistant bacteria in broiler beds can surpass 60%, which could result in a clear risk of environmental pollution [28].

Once in the environment, soil might retain antimicrobials or plants might absorb them, depending on the physicochemical properties of these drugs, as well as on the soil’s properties [25]. In fact, many studies have already shown that plants could absorb these drugs from their environment [1,29,30,31], whereas others have also reported on the phytotoxicological effects that these drugs bring about on crops [32,33,34]. Furthermore, antimicrobial toxicity is not only restricted to plants but also affects aquatic animals, who have presented both acute and chronic manifestations of toxicity [35,36,37,38].

Although the detection and the quantification of antimicrobial residues in feces from farm animals have been previously reported in the scientific literature, we are not aware of any study exploring antimicrobial depletion in poultry droppings following therapeutic treatment regimens (i.e., dose is known). Therefore, our work offers the first stepping stone to building knowledge in this subject. Likewise, it will also be the first study on the detection of tetracyclines, the assessment of their environmental persistence, and the determination of the presence of resistance genes in chicken droppings.

More specifically, this work aimed at studying the depletion of CTC and 4-epi-CTC (its active metabolite) in droppings from broiler chickens that received therapeutic doses of CTC. It also intended to determine the antimicrobial activity of those droppings, as well as observe for the presence of the *tet(A)*, *tet(B)*, and *tet(G)* resistance genes in them. Lastly, it analyzed the susceptibility to tetracyclines of *E. coli* strains that were isolated from these dropping samples. To ensure that these targets were met, we extracted the analyte using solid-phase extraction columns, and then we quantified it by liquid chromatography coupled to mass spectrometry (LC MS/MS). Then, we determined antimicrobial activity via the method of inhibition of bacterial plate growth. Finally, the susceptibility to tetracyclines was assessed using the Kirby–Bauer test, and the presence of resistance genes was detected via a conventional PCR technique.

## 2. Results

### 2.1. In-House Validation of the Analytical Methodology

Specificity, limit of detection (LOD), limit of quantification (LOQ), and linearity were the parameters used for the in-house validation of the analytical method. In the case of specificity, the analysis of blank samples showed no interferences afterwards, thus proving that the method was specific for these analytes. Meanwhile, the LOD was set at a concentration of 20 μg/kg as its signal-to-noise reached at least a 3:1 ratio. The LOQ was defined using the calculated LOD as a starting point and then adding to it 1.64 times the standard deviation of all repetitions. Hence, the LOQ was set at a concentration of 22.5 μg/kg for CTC and of 22.9 μg/kg for 4-epi-CTC. Linearity was assessed by plotting curves (in duplicate), first for concentrations of 20, 40, 60, 80, and 100 μg/kg, and then for concentrations of 200, 400, 800, 1200, and 1400 μg/kg. Only those curves whose R^2^ was greater than 0.99 were accepted.

### 2.2. Depletion of CTC and 4-epi-CTC in Droppings from Broiler Chickens Treated with Tetracyclines

The concentrations of CTC and 4-epi-CTC that were quantified in the droppings samples (from chickens treated with therapeutic doses of tetracyclines) were submitted to a linear regression analysis of the calibration curves in the fortified matrix (R^2^ ≥ 0.99). Our study showed that the concentrations of the analytes declined gradually starting at five days (i.e., first sampling point) up until 25 days after ceasing treatment (i.e., the last sampling point). It is important to highlight that after meeting the withdrawal time for muscle tissue (14 days), our chickens continued to excrete sizable concentrations of these analytes (Table 1).

**Figure 1:**
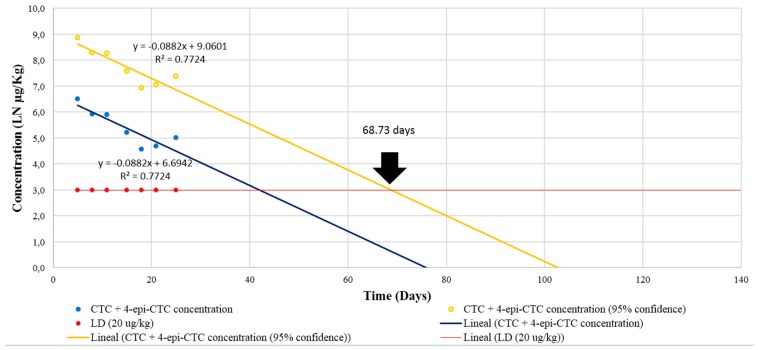
Depletion of CTC and 4-epi-CTC concentrations in chicken droppings (95% confidence level) showing a withdrawal time of 69 days.

As there is currently no withdrawal time set for chicken droppings, we followed the recommendations from the Committee for Veterinary Medicinal Products, European Medicines Agency (CVMP) [39] to calculate one. First, the sample results for the depletion period were plotted as a semilogarithmic scale of concentration against time (Figure 1), then we performed a regression analysis considering a 95% confidence level and determined that the analytes reached concentrations equal to the LOD on day 69 (rounded up to a full day from 68.733) for both CTC and 4-epi-CTC.

### 2.3. Evaluation of Antimicrobial Activity of CTC Residues Present in Chicken Broiler Droppings

**Figure 2:**
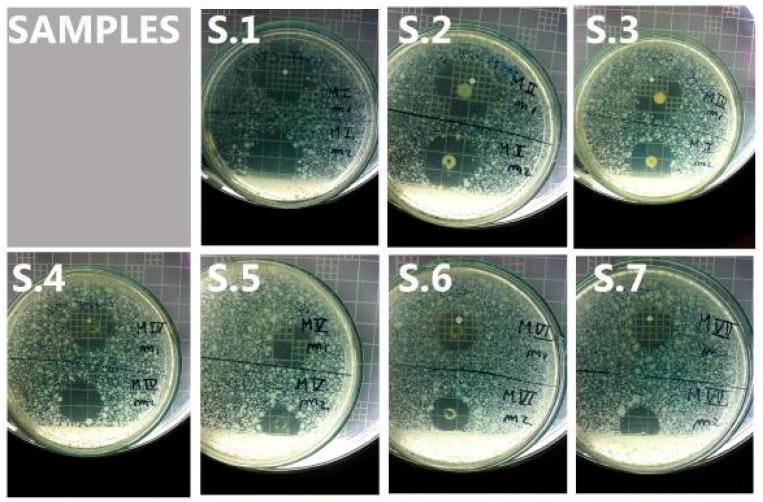
Results from microbiological screening for residues of CTC and 4-epi-CTC in chicken droppings. Plates S1 to S7 represent sampling points 1 to 7. Each sampling point was assessed on duplicate samples (i.e., two cylinders were placed on each plate). Hence, the values for the halo diameter at each sampling point represent the average of these duplicate samples. On average, inhibition halos measured 2.2, 2.2, 2.0, 1.7, 1.4, 1.2, and 1.3 cm, respectively.

All chlortetracycline residues showed active antimicrobial inhibition of bacterial growth at every sampling point, as evidenced by the halos whose diameters were greater than 1.2 cm wide (Figure 2).

### 2.4. Detection of Resistance Genes

**Figure 3:**
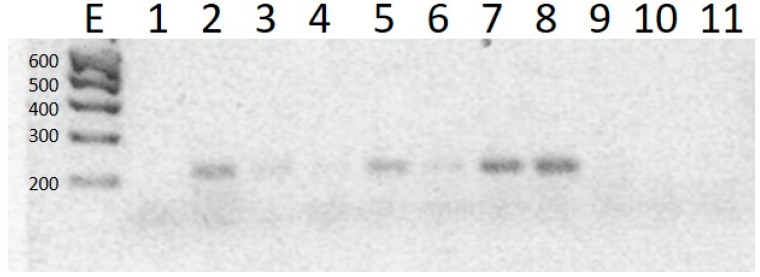
Picture of a 2% Agarose gel, dyed with redGel^®^ stain, representative of PCR products of *E. coli*, using primers for the *tet(A)* gene (210 bp) in droppings from broiler chickens that were treated with therapeutic doses of CTC. Lane E: Molecular weight marker; Lane 1: negative control; Lanes 2 to 8: amplified product of sample treated with chlortetracycline; Lanes 9 to 11: *tet(A)* gene was not expressed and they represent products from blank samples sourced from birds in the control group.

All droppings samples, as well as the control samples, were analyzed by PCR on days 5, 8, 11, 15, 18, 21, and 25 after ceasing treatment with chlortetracycline. Whereas the *tet(A)* and *tet(B)* genes were detected in every treated sample, *tet(G)* was not detected in any of them. Figure 3 shows that the *tet(A)* gene amplified 210 bp bands.

### 2.5. Phenotypic Susceptibility to Tetracyclines

Five *E. coli* strains were isolated for each of the seven sampling time points (i.e., days 5, 8, 11, 15, 18, 21, and 25 after ceasing treatment with chlortetracycline), as well as for all the control groups. However, tetracycline-resistant *E. coli* strains were isolated only from those samples that were collected from birds who were treated with the drug.

## 3. Discussion

Analyzing antimicrobials in animal feces is a noninvasive sampling method that allows for the collection of different kinds of information such as the trends on drug use in farms, the spillover of drugs into the environment, the likely ecotoxicological effects of these, and whether or not the possibility for the emergence of resistant bacteria in the digestive tract of domestic animals exists [40].

Concentrations of CTC and 4-epi-CTC during sampling dates in our study remained high even after the end of the withdrawal period usually recommended in muscle tissue for the commercial pharmaceutical formulation used in this work (14 days). Although concentrations declined gradually up to day 18 after ceasing treatment, they increased again by day 21 and 25. Also, on the last day of sampling, concentrations reached values of 179.5 µg/kg, exceeding the Maximum Residue Limit that has been established for muscle tissue in Commission Regulation (EU) No 37/2010, on pharmacologically active substances and their classification regarding maximum residue limits in foodstuffs of animal origin (100 µg/kg). Such a prolonged elimination period for this drug in chicken droppings could be partly explained by the pharmacodynamics of tetracyclines because CTC undergoes enterohepatic circulation in the same manner as OTC. This cycle limits their bioavailability but ensures that significant concentrations of the drug remain present in the gastrointestinal tract [41]. Nonetheless, it is up for debate on whether or not this would explain that CTC and 4-epi-CTC may be excreted up to 25 days after ceasing a therapy course. In fact, Cornejo et al. [42], reported that they detected OTC and 4-epi-OTC in broiler chicken livers only up to day six after ceasing treatment with those drugs. Such a finding hints that it is indeed unlikely that CTC and 4-epi-CTC residues may remain present in the enterohepatic circulation for 25 days.

Additionally, Berendsen et al. [43] concluded that when OTC is administered to chickens, part of this drug is incorporated into their feathers as they develop, and its concentration in them remains higher than in muscle or liver tissues after the withdrawal period has been fulfilled. This situation was also reported by Cornejo et al. [42], who found that OTC and 4-epi-OTC residues could still be quantified in feathers by day 46 after ceasing treatment. Moreover, withdrawal periods for OTC and 4-epi-OTC in chicken claws have been established at 39 and 54 days, respectively, hence surpassing even the age of slaughter that is currently practiced by the poultry industry [44]. Such evidence suggests that finding these elevated concentrations of CTC and 4-epi-CTC in chicken droppings on the last sampling day (as well as the observation that concentrations increased on day 21 and 25), could be attributed to the systemic recirculation of these residues from feathers and claws. Nevertheless, it would be appropriate to confirm this hypothesis by performing a depletion study that also measures the plasma concentrations of these antimicrobials.

The disk-diffusion method for the inhibition of bacterial growth in agar plates is the official test for the routine assessment of antimicrobial susceptibility in many laboratories of clinical microbiology [45]. To this date, this method has been widely used on several analytical matrices. However, no studies have been published regarding its use for the determination of antimicrobial activity in feces. In the case of the assessment of the susceptibility to tetracyclines in samples of muscle, liver, and kidney tissue, many studies have found that *Bacillus cereus* is the most appropriate species to use for this purpose among all the tetracycline-sensitive bacteria. Specifically, inhibition halos whose diameter ranges between 14 and 15 mm are deemed positive samples [46,47,48]. Therefore, our results indicate that for every sampling date, all samples were positive for tetracycline activity (Table 1). This is consistent with reports from Montforts et al. [49], who found that oxytetracycline and chlortetracycline were excreted unaltered by wethers and young bulls, respectively.

Regarding the detection of resistance genes, our work shows that when chickens were treated with doses of tetracyclines that followed the recommended therapeutic doses and schedules, these drugs generated a selective pressure on the intestinal microbiota that favored bacteria who were carriers of the *tet(A)* and *tet(B)* resistance genes. Considering that these bacteria end up being excreted in droppings, they could contribute with their genes to the environmental resistome, which clearly poses a risk to public health [50].

Nowadays, it is well known that using manure-based fertilizers sourced from domestic animals does contribute to the transferal of resistance genes to soils [51]. Furthermore, Wang et al. [52] have indicated that the application of manure as a fertilizer in soils results in an increased abundance and spread of genes for bacterial resistance to sulfonamides in those soils. Likewise, Wang et al. [53] reported finding ARGs in produce grown in soils that were fertilized with manure. Also, Xie et al. [54,55] reported that chlortetracycline and tetracycline were emergent pollutants, and they showed their potential genotoxicity to the root-meristem cells of wheat (*Triticum aestivum* L.). Naturally then, and bearing in mind that these produce are preferentially consumed raw, these findings are highly important in regards to the risk they pose to public health—a danger that has not been duly appraised by regulatory agencies.

Further compounding the problem, our results indicated that besides the presence of resistance genes in bacteria, they were also phenotypically resistant. These bacteria were being released into the environment in bird droppings; thus, they were likely to contaminate soils, water, and foods, and they were ultimately transferred to other animals and human beings. This chain of events clearly signals an important threat to public health due to the role that tetracyclines play in the treatment of bacterial infections in humans.

## 4. Materials and Methods

### 4.1. Experimental Animals

A total of 60 male, one-day-old chickens from the Ross^®^ 308 broiler strain were housed in cage batteries under controlled environmental conditions (25 ± 5 °C, 50–60% relative humidity). These birds were allowed ad libitum access to water and nonmedicated food.

To ensure the absence of contaminants that may alter our results, both nonmedicated food and bird droppings were tested. In the case of food, the presence of residues of CTC and 4-epi-CTC was tested by liquid chromatography coupled to mass spectrometry, whereas for the droppings, these were tested by PCR to detect the presence of the *tet(A)*, *tet(B)*, and *tet(G)* resistance genes.

Every chicken was randomly assigned to either the experimental or the control group when they turned 14 days old. The experimental group comprised 48 birds and received 50 mg kg^−1^/day of a commercial formulation of 20% chlortetracycline (“Aurofac 200”, Zoetis^®^, Parsippany, NJ, USA) for a period of seven consecutive days. The drug was administered directly in their proventriculus by means of an esophageal catheter to ensure that the birds ingested the full dose. Meanwhile, the control group comprised twelve birds who did not receive the antimicrobial treatment. The size of each group was calculated by following the criteria proposed by the European Medicines Agency in the Note for Guidance EMA/CVMP/SWP/735325/2012 regarding ‘Approach towards harmonization of withdrawal periods’ [39].

Our birds were housed and looked after by following animal welfare recommendations set forth by the Bioethics Committee of the Faculty of Veterinary and Animal Sciences from the University of Chile in its resolution 03-2013 from 11 November 2013. These recommendations were based on Directive 2010/63/EU ‘Regarding the Protection of Animals Used for Scientific Purposes’ [56].

### 4.2. Collection of Samples

Droppings from all birds were collected and pooled for each sampling day (i.e., days 5, 8, 11, 15, 18, 21, and 25 after ceasing treatment) in both the experimental and control groups. One gram of each pool was used to isolate *E. coli* bacteria in MacConkey agar plates (incubated at 37 °C for 24 h). From these plates, five colonies were selected to perform susceptibility tests. The remaining samples were packed individually in plastic bags and stored at −80 °C while waiting for further analysis.

### 4.3. Quantification of CTC and 4-epi-CTC in Droppings

#### 4.3.1. Reagents and Equipment

Chlortetracycline was sourced from Dr. Ehrenstorfer Gmbh (LGC Standards, Middlesex, UK), Sigma Aldrich (Merck, Darmstadt, Germany) or a similar manufacturer, and 4-epi-Chlortetracycline was sourced from Acros Organics^®^ (Thermo Fisher Scientific, Waltham, MA, USA). Both analytes were of certified laboratory-standard quality. Water, methanol, and acetonitrile were of HPLC-grade and sourced from Millipore (Merck, Darmstadt, Germany) or a similar manufacturer. Deuterated tetracycline (TC-d_6_) was used as an internal standard and sourced from Toronto Research Chemicals (Toronto, ON, Canada). All other reagents were of analytical-grade quality and sourced from Fisher (Thermo Fisher Scientific, Waltham, MA, USA), Merck (Merck, Darmstadt, Germany), or a similar company.

First, primary stock solutions were prepared by dissolving 500 ng/mL of either CTC or 4-epi-CTC, and 1000 ng/mL of TC-d_6_ in methanol. Then, working solutions that were suitable to spike the blank samples were prepared by diluting stock solutions in methanol down to a concentration of 2.5 ng/mL for CTC and 4-epi-CTC, and of 20 ng/mL for TC-d_6_. All of these solutions were then stored at −80 °C.

#### 4.3.2. Sample Preparation, Extraction and Clean-Up

All samples were homogenized with a wooden stick before weighing in 2 g of each one in 50 mL polypropylene tubes. Every sample was fortified with the internal standard TC-d_6_, whereas the tubes that were meant to be blank control samples were also fortified with CTC and 4-epi-CTC.

To extract the analytes from the matrix, 4 mL of EDTA–McIlvaine buffer solution and 1 mL of acetonitrile were added as solvents to each tube, and the mixture was homogenized for 10 min in a tube shaker. Then, samples were centrifuged at 1000× *g* for 10 min and the supernatant was filtered using glass wool. This extract was then diluted by adding 13 mL of EDTA–McIlvaine solution and passed through OASIS™ HLB^®^ solid-phase extraction columns (Waters Corp., Milford, MA, USA). These columns had been previously conditioned with 5 mL of methanol and 5 mL of HPLC-grade water flowing at a rate of 1 mL min^−1^. After passing the extract through the columns, it was eluted with 5 mL of methanol, and the resulting sample was subsequently evaporated at 40–50 °C under a soft flow of nitrogen.

The final step before proceeding with the chromatographic analysis was to reconstitute these concentrated solutions by pouring them along with 200 µL of methanol and 300 µL of HPLC-grade water into Eppendorf tubes. These tubes were then centrifuged at 17,369× *g* for 10 min, and the supernatant was passed through Millipore (Merck, Darmstadt, Germany) filters while they were being transferred to glass vials.

#### 4.3.3. Instrumental Analysis

Samples were analyzed using an Agilent^®^ Series 3200 liquid chromatograph (Agilent Technologies, Santa Clara, CA, USA) coupled to an ABSCIEX^®^ API 4000 mass spectrometer (SCIEX, Framingham, MA, USA) and a Sunfire^®^ C18 (Waters Corp., Milford, MA, USA) chromatographic column of 3.5 µm and 2.1 × 150 mm.

The analytes were chromatographically separated using a mobile phase gradient of 0.1% formic acid in water (Phase A) and 0.1% formic acid in methanol (Phase B). The flow rate for this mobile phase was set at 0.2 mL min^−1^, the injection volume was set at 25 µL, and the column temperature was set at 30 °C. 

The criteria to identify CTC and 4-epi-CTC was the detection of precursor ions with masses of 479.0 Da. Similarly, precursor ions of mass 451 Da were regarded as indicative of TC-d_6_. Meanwhile, fragmented ions had masses of 444.0, 154.0, and 416.0 Da for CTC, 4-epi-CTC, and TC-d_6_, respectively.

As for the chromatographic integration of the samples, we used the Analyst^®^ version 1.6.2 software (SCIEX, Framingham, MA, USA).

### 4.4. Determination of Antimicrobial Activity

In parallel to the chromatographic analysis, droppings from birds treated with tetracycline were tested for antimicrobial activity of CTC residues by using a specific bacterial growth inhibition method based on methodologies that were published independently by Pikkemaat et al. [57], and Gaudin [58].

#### 4.4.1. Preparation of Culture Media

A suspension of *Bacillus cereus* spores from the ATCC^®^ 11778^TM^ strain (American Type Culture Collection, Manassas, VA, USA), at a concentration of 3 × 10^8^ cfu/mL was cultured in a Difco^TM^ Antimicrobial Medium 8 sterile broth (Becton, Dickinson and Company; Franklin Lakes, NJ, USA). This broth was adjusted to pH 6 and kept warm at 50 °C for 15 min, before drawing 10 mL of this inoculum to sow bacteria in Petri dishes. These plates were closed and left to cool down on a flat surface afterwards.

#### 4.4.2. Extraction of Antimicrobials from Samples and Preparation of Plates for Growth Inhibition Assessment

Besides the depletion study, antimicrobial residues were also extracted for assessment of their activity via microbiological methods. In this case, the extraction procedure began by weighing in 5 g of droppings in a 50 mL polypropylene tube, then adding citric–acetone buffer solvent. This mixture was homogenized and sonicated for 15 min twice. Then, samples were centrifuged at 1000× *g* for 15 min, and 200 µL from the resulting supernatant was poured in metallic cylinders (in duplicate) placed on top of the agar plates that were previously inoculated with *Bacillus cereus* bacteria. These plates were incubated at 30 °C for 18 h and read by measuring the diameter of the inhibition halo using a precision Vernier caliper. A dropping sample was regarded to be positive for the presence of active antimicrobial residues when the diameter of the inhibition halos was greater than or equal to 1.2 ± 0.2 cm.

### 4.5. Detection of Genes Conferring Resistance to Tetracyclines

Droppings samples from both the experimental and the control groups were tested for the presence of resistance genes. The DNA from these droppings samples was extracted using a QIAamp^®^ Mini 51,604 kit (QIAGEN, Hilden, Germany), according to the operating instructions provided by the manufacturer. The DNA of the *Escherichia coli* ATCC 25922 strain was also extracted and used as a negative control sample.

Once the DNA was extracted, each resistance gene was amplified by a conventional PCR technique using an ESCO^®^ thermocycler (Esco Micro Pte. Ltd., Singapore). Table 2 describes the primers sequence, the annealing temperatures, and the size of each product for every resistance gene. Strains of isolated *Escherichia coli* were sequenced and those presenting the specific resistance gene were used as positive controls. Once the PCR amplification was complete, genes were separated by electrophoresis in 1% agar gel at 100 millivolts for 40 min in 1x Tris base, acetic acid, and EDTA buffer (TAE buffer). The molecular marker had a molecular weight of 100 bp. After completing this process, the gel was dyed using ViSafe^®^ Red Gel^®^ stain (Vivantis Technologies Sdn. Bhd., Subang Jaya, Malaysia) and observed under UV light in a BIOTOP^®^ BIOSENS SC750 gel documentation system (Shanghai Bio-Tech Co., Ltd., Shanghai, China).

### 4.6. Determination of Susceptibility to Tetracyclines in Isolates of E. coli Bacteria

The agar diffusion test (Kirby–Bauer) was used to assess the phenotypic resistance to tetracyclines in *E. coli* bacteria that were isolated from the droppings of treated birds, as it is recommended by Clinical Laboratory Standards Institute (CLSI) guidelines [60]. Testing was performed using Oxoid^®^ TE discs (30 µg/disc), and *E. coli* ATCC 25922 bacteria were used for quality control purposes.

## 5. Conclusions

The methodology used in this work allowed us to not only determine CTC and 4-epi-CTC residue concentrations in broiler chicken droppings, but also to assess their antimicrobial activity, as well as to detect both the presence of resistance genes and strains of resistant bacteria in those samples. Our results showed that tetracyclines were present at high concentration levels for up to 25 days after ceasing treatment with chlortetracycline, and that *tet(A)* and *tet(B)* genes were highly prevalent in those samples.

This work is highly significant because tetracyclines are one of the most commonly used classes of antimicrobial drugs within the scope of veterinary practice. Consequently, it could be anticipated that major quantities of these active antimicrobial residues, the strains of resistant bacteria, and the resistance genes are currently being released in the environment. These findings might contribute to the expansion of scientific knowledge in regards to the environmental impact of tetracyclines and the risk that the presence of antimicrobial drug residues in bird droppings pose to public health. Furthermore, they could also aid to rethink what the final end should be for all droppings that are collected from animals that had been treated with antimicrobials, as well as what measures could eventually be implemented to improve the management of these kinds of byproducts.

## Figures and Tables

**Table 1:** Chlortetracycline (CTC) and 4-epi-CTC concentrations, assessment of antimicrobial activity, and detection of resistance genes in broiler chicken droppings by sampling point.

Sampling Point	Days after Treatment	Chicken Age (in Days)	Antimicrobial Activity in Broiler Chicken Droppings	Concentrations of CTC and 4-epi-CTC (μg/kg) in Broiler Chicken Droppings	PCR		
					*tet* A	*tet* B	*tet* G
Sample 1	5	25	p	665.8	+	+	-
Control 1			a	<LOD	-	-	-
Sample 2	8	28	p	368.2	+	+	-
Control 2			a	<LOD	-	-	-
Sample 3	11	31	p	258.4	+	+	-
Control 3			a	<LOD	-	-	-
Sample 4	15	35	p	136.9	+	+	-
Control 4			a	0	-	-	-
Sample 5	18	38	p	106.5	+	+	-
Control 5			a	<LOD	-	-	-
Sample 6	21	41	p	112.0	+	+	-
Control 6			a	<LOD	-	-	-
Sample 7	25	45	p	179.5	+	+	-
Control 7			a	<LOD	-	-	-

p: antimicrobial activity is present; a: antimicrobial activity is absent; +: Resistance gene is present; -: Resistance gene is absent.

**Table 2:** Primers and annealing temperatures used in PCR assays for each resistance gene.

Gene	PCR	Primer Sequence (5′-3′)	Product Size (bp)	Annealing Temperature (°C)	Resistance Phenotypes
*tet*(A)	F	gctacatcctgcttgccttc	210	56.2	TET
	R	catagatcgccgtgaagagg		55.1	
*tet*(B)	F	ttggttaggggcaagttttg	659	53.5	TET
	R	gtaatgggccaataacaccg		53.9	
*tet*(G)	F	gctcggtggtatctctgctc	468	57.3	TET
	R	agcaacagaatcgggaacac		55.5	

TET: Tetracyclines; Reference: Ng et al. [59].
